# Supplementary material for: Factors associated with stroke among adult patients with hypertension in Ayder Comprehensive Specialized Hospital, Tigray, Ethiopia, 2018: A case-control study
Source: PLoS One. 2020 Feb 13;15(2):e0228650. doi: 10.1371/journal.pone.0228650 (PMC7018071; doi:10.1371/journal.pone.0228650)
Supplement: S1 Appendix — (DOCX) [file pone.0228650.s002.docx]

1. Consent form.

Good morning/ afternoon?

My name is _____________________. I am working as a data collector on behalf of Haftea Hagos who is currently working by Adult Health Nursing at Mekelle University, College of Health Sciences and School of nursing

**Aim of the study**

The purpose of this research project is to assess factors associated with stroke among patient with hypertension.

**Benefit and risks**

Even though there are no immediate benefits from the study to the study population, the findings of the study will help for future planning of improvement of health care provider and patients and providing effective care. There is no risk to the participants of this study except the time you will spend for interview which will take about 30 minutes.

**Confidentiality**

I would like to assure you that your name will not be mentioned in the questionnaire and the information that you will give me will be kept confidential and only used for research purpose and you are kindly asked to give real information honestly. And if you do not want to participate you have full right to refuse at any time. But the information that you will give me is quite useful to achieve the objective of the study. Your participation is voluntary and there is no effect on services that you or any member of your family for your refusal to participate. If you have any question regarding to the study you can contact by 0914436962 or hafteahagos2@gmail.com

**Consent form**

I confirm that I have received verbal information. I have understood the subject information of the study and had the opportunity to discuss the study and questions. I agree to participate in this research study. **Respondent’s signature_________________________________**

Date of interview: _______________ Time started: _______ Time finished: _________

Interviewer Name_________________________Signature___________Date____________

Supervisor’s name ________________ signature ________

Results of interview questionnaire

1. Completed

2. Refused

3. Partially completed

**II. English version questionnaire**

1. **Socio-demographic data**

| **Questions** | | **Response** |
| --- | --- | --- |
| 1 | Sex | 1. Male 2. Female |
| 2 | Age | In years_____________ |
| 3 | Religion | 1. Christian orthodox 2. Muslim 3. Catholic 4. Other specify ____________ |
| 4 | Ethnicity | 1. Tigray 2. Amhara 3. Afar 4. Other specify___________ |
| 5 | Marital status | 1. Married 2. Single 3. Divorce 4. Widowed |
| 6 | Level of education | 1. No formal education 2. Primary school 3. Secondary school 4. Diploma 5. University degree and more |
| 7 | Occupation | 1. Farmer 2. House wife 3. Government employee 4. Non-Government organization employee 5. Self-employee 6. Other(s) [Specify]:_______ |
| 8 | Residency | 1. Rural 2. Urban |
| 9 | Average family income per month | In birr _________ |
| 10 | Do you have family history of stroke? | 1. Yes 2. No |

1. **Medical information**

| No | Questions | Response |
| --- | --- | --- |
| 1 | How long has it been since you were diagnosed with hypertension? | _________ |
| 2 | What is your frequency of follow-up? | 1. Monthly 2. Every 2 monthly 3. Every 3 monthly 4. Other specify _________ |
| 3 | Have you ever lost to your follow-up (as per the health professionals appoint you)? | 1. Yes 2. No |

1. **Behavioral risk factors for stroke**

| **Questions** | | **Response** | **Skip** |
| --- | --- | --- | --- |
| 1 | \| Have you ever smoked cigarettes? \| \| --- \| | 1. Yes 2. No | If No to Q 3 |
| 2 | Have you smoke cigarette after you diagnosed hypertensive | 1. Yes 2. No |  |
| 5 | Have you ever drunk Alcohol? | 1. Yes 2. No | If No to Q 9 |
| 6 | Have you drink alcohol after you diagnosed hypertensive? | 1. Yes 2. No |  |
| 7 | Types of alcohol you drink | _________  _________  _________ |  |
| 8 | Average amount of alcohol you drink/week | _________ |  |
| 9 | Do you reduce salt amount in diet | 1. Yes 2. No |  |
| 10 | Did you eat diet high in fat such as Fatty meal and animal product? | 1. Yes 2. No |  |
| 11 | How many days do you do regular physical exercise in a week? | In day ___________ |  |
| 12 | On average how much time do you spend during those exercise in a typical day? | In hours___________ |  |

1. **Medication adherence (MMAS)**

| **Questions** | | **Yes** | **No** |
| --- | --- | --- | --- |
| 1 | \| Do you sometimes forget to take your pills? \| \| --- \| |  |  |
| 2 | People sometimes miss taking their medications for reasons other than forgetting. Thinking over the past two weeks, were there any days when you did not take your medicine? |  |  |
| 3 | Have you ever cut back or stopped taking your medicine without telling your doctor because you felt worse when you took it? |  |  |
| 4 | When you travel or leave home, do you sometimes forget to bring along your medicine? |  |  |
| 5 | Did you take all your medicine yesterday? |  |  |
| 6 | When you feel like your symptoms are under control, do you sometimes stop taking your medicine? |  |  |
| 7 | Taking medicine every day is a real inconvenience for some people. Do you ever feel hassled about sticking to your treatment plan? |  |  |
| 8 | How often do you have difficulty remembering to take all your medicine?  a. Never/rarely b. Once in a while  c. Sometimes d. Usually e. All the time |  |  |

1. **Physical measurements**

| 1 | Current Blood Pressure | ___________mmHg |
| --- | --- | --- |
| 2 | Current Anthropometric measurement | Height __________cm  Weight _________kg  Waist circumference ____cm  BMI ­­­­­­­­­­­__________Kg/m^2^ |

1. **Data abstraction format from chart**

| 1 | Diagnosis | 1. Hypertension 2. Stroke and history of hypertension |
| --- | --- | --- |
| 2 | Stroke status of the patient? | 1. New 2. Recurrent |
| 2 | What subtype of stroke was diagnosed? | 1. Ischemic 2. Hemorrhagic stroke |
| 3 | Which images method was performed to diagnose stroke? | 1. CT-scan 2. MRI 3. Clinical |

| 1 | Blood Pressure (before and during first attack for stroke and before and during data collection for controls ) | 1. ­­­___________mmHg­­­­ 2. ___________mmHg 3. ____________mmHg(during fist attack of stroke for cases and data collection for controls ) |
| --- | --- | --- |
| 2 | Anthropometric measurement (nearest to first stroke for cases and nearest to data collection period for controls) | Height _____________cm  Weight _____________kg  Waist circumference _______cm  BMI _____________Kg/m^2^ |
| 3 | Cholesterol lab. results | Total cholesterol___________________  HDL-cholesterol___________________  LDL-cholesterol___________________  Triglycerides______________________ |
| 4 | Fasting/random glucose level | _________________________________ |

**If you have any question you are welcome**

Thank you for your participation

1. **ናይ ተሳተፍቲ መረጃ መሰብሰቢ ቅጥዒ ብ ትግርኛ**

Code________________

Date ________________

MRN________________

**አብ መቐለ ዩኒቨርሲቲ ጥዕና ሳይንስ ኮሌጅ**

**ነርሲንግ ትምህርቲ ክፍሊ**

አብ ዓይደር ኣጣቓላሊ ስፔሻላይዝድ ሆስፒታል እናተመላለሱ ንሉዑል ፀቕጢ ደምን ወቕዒ ሓንጎልን ክትትል ዝገብሩን ደቂሶም ዝሕከሙ ወቕዒ ሓንጎል ተሓከምትን ዝተዘጋጀወ ቃለ መሕትት ፡፡

ጥዕና ይሃበለይ፡ ስመይ----------------ይባሃል ኣነ መረዳእታ ሰብሳቢ ኮይነ እዚ መረዳእታ ዝእክቦ ሃፍቴ ሓጎስ ኣብ መቐለ ዩኒቨርሲቲ ጥዕና ሳይንስ ኮሌጅ ነርሲንግ ትምህርቲ ክፍሊ ናይ ድሕረ ምረቓ ትምህርቲ ብ ኣዳልት ሄልዝ ነርሲንግ ናይ 2ይ ዲግሪ ተመራቂ ተማሃሪ ናይ መመረቂ ፅሕፎም ንምድላው ንክሕግዞም እዩ፡፡

**ናይዚ ፅንዓት ዕላማ**

መንቀሊ ሕማም ወቕዒ ሓንጎል ልዑል ፀቕጢ ደም ኣብ ዘለዎም ሕሙማን ንምፍላጥ የክእል፡፡

**ጥቅማጥቅሚ ናይዚ መፅናዕቲ**

ምንም እንኳ ካብዚ ፅንዓት ፈጣን ዝኮነ ጥቅሚ ኣይሃልዎ ናዚ ፅንዓት ውፅኢት ግና ልዑል ፀቕጢ ደም ንዘለዎም ሕሙማን ክገብርዎ ዘለዎም ጥንቃቐን ንምፍላጥ የክእል፣ብተወሳኪ ኣብ ጥዕና ብዓልሞያታት ዘሎ ኣባሃህዋ ግልጋሎት ዘሎ ክፈተት ንምስትክካል ዕድል ይህብ፡፡

**ጎናዊ ጉድኣት**

ብዘይካ ን ከባቢ 30 ደቒቓ ን ቃለ ማሕተት ዝከውን ግዜ ምጥፋእ ኣብዚ መፅናዕቲ ብምስታፍ ዝመፅእ ምንም ጉድኣት የለን

**ምሰጢር ምሕላው**

ዝጥይቆም ሕቶታት በቐሊሉ ክመልሰዎም ዝክእሉ እየም ስለዚ ሓሳቦም ብነፃነት ንክክእሉ ሰሞም ምፅሓፍ ኣየድልን፡፡ነገር ግን ስለናቶም/ን ሓቀኛ ዝኮነ መልሲ ኣድላያይ እዩ፡፡ ንዝህበዎ መረዳእታ/ሓበሬታ ሚስጥራዊነቱ ዝተሓለወ እዩ፡፡እዚ ቃለመሕትት ዝምልኣሉ ፍቓደኛታት ንዝኮኑ ሰባት ጥራሕ እዩ፡፡ምስታፍ ፍቃደኛ እንተዘይኮይኖም/ነን ኣብ ዝኮን ግዘ ናይ ሙቁራፅ ትክእሉ ኢኩም/ክን፡፡ንዝህቡና ሓበሬታ/መረዳእታ ፀገሙ ንምፍታሕ ኣብዝግበር ፃዕሪ ዓብዪ ጥቕሚ አለዎ ፅንዓቱ ዝተመልከተ ሕቶ እንተሃልይዎም በዚ ስለኪ ቁፅሪ ናብ ሃፍቴ ሓጎስ ደዊሎም ምጥያቐ ይክእሉ፡፡ ስልክ ፡-0914436962ወይ ብ ኢሜል፡ hafteahagos2@gmail.com

ናይ ስምምዕነት ውዕል

እዚ ፅንዓት ዘፅንዖ ግለሰብ ብዘረደአኒ መሰረት ብፍቓደይ ኣብዚ ፅንዓት ንምስታፍ

ፍቃደኛ እየ

ናይ ተሓታታይ ፌርማ…………………………

ዝተሐተተሉ ዕለት-----------------ዝተጀመረሉ ሰዓት-------------ዝተወደአሉ ሰዓት----------------

ናይ ሐታታይ ሽም---------------------------------------------------- ፊርማ-----------------

ሽም ተቆፃፃሪ-------------------- ፊርማ-----------------

ውፅኢት ቃለ መሕትት

1. ዝተማልአ

2. ዝተነፀገ

3. ዘይተማለአ

1. **ማሕበራዊ መረዳእታ**

| ሕቶታት | | መልሲ ተሓታቲ |
| --- | --- | --- |
| 1 | ፆታ | 1. ተባዕታይ 2. ኣንስታይ |
| 2 | ዕድመኹም/ክን ክንደይ እዩ? | ብዓመት­­­­­­­­­­­­­­------ |
| 3 | እትኽተልዎ/ኦ ሃይማኖታዊ እምነት | 1. ኦርቶዶክስ 2. እስልምና 3. ካቶሊክ 4. ካሊእይገለፅ__________ |
| 4 | ብሄር | 1. ትግራይ 2. ኣምሓራ 3. ዓፋር   4. ካሊእ(ይገለፅ)------------- |
| 5 | ኩነታትሓዳር | 1. በዓል/ቲ ሓዳር 2. ዘይእተወ/ት 3. ዝተፋተሐ/ት 4. መዋስብቲ ዝሞተቶ/ታ |
| 6 | ደረጃ ትምህርቲ | 1. ሰሩዕ ትምህርቲ ዘይተማሃረ 2. ቀዳማይ ደረጃ 3. ካልኣይ ደረጃ 4. ዲፕሎማን 5. ዩኒቨርስትን ልዕሊኡን |
| 7 | ስራሕ | 1. ሓረስታይ 2. ናይገዛ ስራሕተኛ 3. ሰራሕተኛ መንግስቲ 4. ሰራሕተኛ ዘይመንግስታዊ ትካል   5. ብውልቀ ስራሕ ዝመሓደር 6. ከሊእ ይገለፅ________ |
| 8 | ዝነብርሉ ቦታ | 1. ገጠር 2. ከተማ |
| 9 | ናይ ቤተ ሰብ ብማእከላይ ወርሓዊ ኣታዊ ብብር | ­­­­­­­_________ |
| 10 | ካብ ብተስበኩም ወቕዒ ሓንጎል ሓሚሙ ዝፈልጥ ኣሎ ዶ | 1. እወ 2. የለን |

1. **ምስ ሕማሙ ዝተታሓሓዙ ጉዳያት**

| ቁፅሪ | ሕቶታት | መልሲ |
| --- | --- | --- |
| 1 | ልዑል ፀቕጢ ደም ኣለዎም ካብ ዝብሃል ክንደይ ገይሮም/ረን |  |
| 2 | በብክንደይ ክትተል ይገብሩ/ራ | 1.ብወርሒ 2. ብ 2 ወርሒ 3. ብ 3 ወርሒ  ከሊእይገለፅ___________ |
| 3 | ጥዕና ባዓል ሞያ ብዝኣዘዞም/ን መሰረት ክትትል ኣቃሪፆም/ን ዶ ይፈል/ጣ | 1. እወ 2. ኣይፈልጥን |

1. **ምስ መነባብሮ ዝተታሓሓዙ ጉዳያት**

| 1 | ሽጋራ ኣትኪኩም/ን ትፈልጡ/ጣ ዶ | 1. እወ 2. ኣየትክክን |
| --- | --- | --- |
|  | ልዑል ፀቕጢ ደም ምህላዎም ካብ ዝፈልጡ ሽጋራ ኣተኪኩም/ን ዶ? | 1. እወ 2. ኣየትክክን |
| 2. | ኣልኮል ሰቲካ/ን ዶ ትፈልጥ/ጣ | 1. እወ 2. ኣይሰትን |
|  | ልዑል ፀቕጢ ደም ምህላዎም ካብ ዝፈልጡ ኣልኮላዊ መስተ ሰቲኩም/ን ዶ? | 1. እወ 2. ኣይሰትን |
|  | ሰሙናዊ እትሰትይዎ/ኦ መጠን ኣልኮላዊ መስተ ክንደይ ይኸውን?(ዓይነትን መስተን መዓልታዊ እትሰትይዎ መጠን ኣልኮልን ጥቐሱ) | ______________ |
| 4 | ኣብ ምግቢ ጨው ቀኒስኩም/ን ዶ | 1. እወ 2. ኣይዳለውን |
| 5 | ስብሒ ዝበዘሖ ስጋ ወይ ድማ ዝኾነ ውፅኢት እንስሳት ትጥቀሙ ዶ | 1. እወ 2. ኣይጥቀምን |
| 6 | ሱሩዕ ኣካላዊ ምንቅስቓስ ኣብ ሰሙን ንኽንደይ መዓልቲ ትሰርሑ/ሓ? | በዝሒ መዓልቲ ____ |
|  | ብማእከላይ ኣብ ትሰርሕሉ እዋን ንክነደይ ዝኣክል ይሰርሑ/ሓ | ብ ሳዓት­­­­­­­­­ ____________ |

1. **ኣጣቃቅማ መድሓኒት ዝተመልከተ**

| ሕቶታት | | እወ | ኣይፋል |
| --- | --- | --- | --- |
| 1 | \| ሓደ ሓደ ግዘ መድሓኒትኩም ምውሳድ ረሲዕኩም/ን ዶ ትፈልጡ/ጣ? \| \| --- \| |  |  |
| 2 | ሰባት ሓደ ሓደ ግዘ ብምርሳዕ ዘይኮነስ ብከሊእ ምክንያት መድሓኒት ምውሳድ የቃርፁ እዮም ንዝሓለፉ ክልተ ሰሙን ብምስትዋስ መድሓኒት ዘይወሰድሎም/ዳሎም ማዕልትታት ኣለዉ ዶ ? |  |  |
| 3 | ብዘይ ናይ ዶክተር ትእዛዝ ምልክታት እቲ ሕማም ዝባኣሱ እንትመሰሉም ድሓኒቶም ውሳድ ኣቓሪፆም/ን ዶ ይፈልጡ/ጣ ? |  |  |
| 4 | ሓደ ሓደ ግዘ መገሻ /ካብ ገዘኦም እንትወፅኡ መድሓኒት ምውሳድ ረሲዖም/ን ዶ ይፈልጡ/ጣ? |  |  |
| 5 | ትማሊ ኩሉ መድሓኒቶም/ን ዶ ወሲዶም/ን? |  |  |
| 6 | ምልክታት እቲ ሕማም ዝጠፍኡ እንትመሰሉም መድሓኒት ምውሳድ ኣቓሪፆም/ን ዶ ይፈልጡ/ጣ? |  |  |
| 7 | መድሓኒት ኩሉ ግዘ ምውሳድ ንዝተወሰኑ ሰባት የሰልችዮም እዩ ንሶም/ሰን መድሓኒት ምውሳድ ሰልችኩም ፕሮግራምኩም/ን ኣዛቢዕኩም ዶ ትፈልጡ ? |  |  |
| 8 | መድሓኒቶም/ን ንምውሳድ ምዝካር ይሽገሩ/ራ ዶ ?  1. ብፉፁም 2. ሓሓሊፉ 3. ሓደ ሓደ ግዘ 4. መብዛሕትኡ ግዘ 5. ኩሉ ግዘ |  |  |

1. **መጠን ፀቕጢ ደምን ሰውነትን**

| 1 | መጠን ፀቕጢ ደም | ___________ሚ.ሜ ሜሪኩሪ |
| --- | --- | --- |
| 2 | መጠን ሰውነት | ዓቐንቁመት____ሴ.ሜ ዓቐንክብደት_______ኪ.ግ  መጠንዙርያማዕጥቖ_______ሴ.ሜ |

1. **ካብ ካርዲ ዝርከቡ ሓበሬታት**

| **ምስ ሕማሙ ዝተታሓሓዙ ጉዳያት** | | | |
| --- | --- | --- | --- |
| 1. | እቲ ስብ ሐዚ ዘለዎ ሕማም | 1. ወቅዒ ሓንጎልን ልዕል ፀቕጢ ደም 2. ልዕል ፀቕጢ ደም | |
| 2. | ዓይነት ወቅዒ ሓንጎል | 1. ኢሰከሚክ ስትሮክ 2. ሄሞረጂክ ስትሮክ | |
| 3. | ወቅዒ ሓንጎል | 1. ሓዲሽ 2. ንክትትል ዝመፀ | |
| 4 | ብምንታይ ዓይነት መሳርሒ ተራእዩ/ን | 1. ሲቲ ስካን 2. ኤምኣርኣይ   3.ብመሰረት ምልክታት | |
|  | | | |
| 1 | መጠን ፀቕጢ ደም ኣብ ዝሓለፉ ክትትላት | | 1. __________ሚ.ሜ ሜሪኩሪ 2. ___________ሚ.ሜ ሜሪኩሪ 3. _________ሚ.ሜ ሜሪኩሪ |
| 2 | መጠን ሰውነትን | | ዓቐንቁመት____ሴ.ሜ ዓቐንክብደት_______ኪ.ግ  መጠንዙርያማዕጥቖ_______ሴ.ሜ |
| 3 | ኮለስተሮል መጠን ውፅኢታት ምስ ማዕልቶም ይገለፅ | | Total cholesterol___________________  HDL-cholesterol___________________  LDL-cholesterol___________________  Triglycerides______________________ |
| 4 | ኣብ ደም ዘሎ መጠን ሽኮር | | ----------------------- |

**ብምስታፍኩም ካብ ልቢ የመሰግን**
