# Supplementary material for: Factors associated with stroke among adult patients with hypertension in Ayder Comprehensive Specialized Hospital, Tigray, Ethiopia, 2018: A case-control study
Source: PLoS One. 2020 Feb 13;15(2):e0228650. doi: 10.1371/journal.pone.0228650 (PMC7018071; doi:10.1371/journal.pone.0228650)
Supplement: Attachment — Submitted filename: renamed_3b9c1.docx [file pone.0228650.s003.docx]

**Manuscript #:**  PONE–D- 31261

**Title:** Factors associated with stroke among Adult patients with Hypertension in Ayder Comprehensive Specialized Hospital, Tigray, Ethiopia, 2018. A Case Control study.

**Review:**

1. The article is well written. However, since the authors are working on the factors associated with stroke, a range of the age group taken (Minimum & maximum age in adults) instead of the mean age would be more informative to further identify the incidence of stroke, whether more common in young adults or older adults.
2. The inclusion of some more parameters like Gender, Socioeconomic status, dietary habits (Consumption of more Fatty or non fatty food), profession (Stressful/ non stressful), life style (Sedentary/ Active), & family history will make this study and identification of factors more meaningful.
