# Supplementary material for: Factors associated with stroke among adult patients with hypertension in Ayder Comprehensive Specialized Hospital, Tigray, Ethiopia, 2018: A case-control study
Source: PLoS One. 2020 Feb 13;15(2):e0228650. doi: 10.1371/journal.pone.0228650 (PMC7018071; doi:10.1371/journal.pone.0228650)
Supplement: Attachment — Submitted filename: Response to Reviewers.docx [file pone.0228650.s004.docx]

**Response to Reviewers**

**PONE-D-19-31261**

**Title: Factors associated with Stroke among Adult Patients with Hypertension in Ayder Comprehensive Specialized Hospital, Tigray, Ethiopia, 2018: A case-control study**

**PLOS ONE**

**Point by point response to Reviewers Comments:-**

| **Sr. No.** | **Reviewers' Comments** | **Response to Comments** |
| --- | --- | --- |
|  | **Academic Editor comments** |  |
| 1 | Please ensure that your manuscript meets PLOS ONE's style requirements, including those for file naming. The PLOS ONE style templates can be found at http://www.plosone.org/attachments/PLOSOne_formatting_sample_main_body.pdf and <http://www.plosone.org/attachments/PLOSOne_formatting_sample_title_authors_affiliations.pdf> | We accept the comment and we made correction accordingly. |
| 2 | In your Methods section please provide a rationale for excluding pregnant women. | Pregnant women were excluded because:   1. Hypertensive mothers increases the risk of stroke during pregnancy due to the pregnancy itself increase the chance of stroke because of physiological effect (hormonal change) so, this physiological change can affect the our result. 2. Anthropometric measurement(weight) is difficult in pregnant mother in order to calculate body mass index (BMI) of the participant. |
| 3 | Please provide an amended Funding Statement that declares *all* the funding or sources of support received during this specific study (whether external or internal to your organization) as detailed online in our guide for authors at <http://journals.plos.org/plosone/s/submit-now>. | The crossponding author gets fund from Mekelle University (from internal organization) but the funder had no role in study design, data collection and analysis, decision to publish, or preparation of the manuscript." |
| 4 | PLOS requires an ORCID iD for the corresponding author in Editorial Manager on papers submitted after December 6th, 2016. Please ensure that you have an ORCID iD and that it is validated in Editorial Manager. | I have created ORCID iD and validated in the editorial manager. |
| 5 | Your ethics statement must appear in the Methods section of your manuscript. If your ethics statement is written in any section besides the Methods, please move it to the Methods section and delete it from any other section. Please also ensure that your ethics statement is included in your manuscript, as the ethics section of your online submission will not be published alongside your manuscript | We corrected it. Ethical statement is now in methods section |
| 6 | Please include your tables as part of your main manuscript and remove the individual files. Please note that supplementary tables (should remain/ be uploaded) as separate "supporting information" files | We accepted your comment. Now tables are within the main manscript and individual tables are removed. |
| 7 | Please include captions for your Supporting Information files at the end of your manuscript, and update any in-text citations to match accordingly. Please see our Supporting Information guidelines for more information: <http://journals.plos.org/plosone/s/supporting-information> | We accept the comments and sugestions. We made correction accordingly |
| 8 | We note that you have stated that you will provide repository information for your data at acceptance. Should your manuscript be accepted for publication, we will hold it until you provide the relevant accession numbers or DOIs necessary to access your data. If you wish to make changes to your Data Availability statement, please describe these changes in your cover letter and we will update your Data Availability statement to reflect the information you provide. | We changed our idea and the data is now available in "supporting information" files |
| 9 | We suggest you thoroughly copyedit your manuscript for language usage, spelling, and grammar. If you do not know anyone who can help you do this, you may wish to consider employing a professional scientific editing service. | We accept the comment and we made corrections regarding the grammatical and punctuation errors throughout the whole document. |
|  | **Reviewer#1** |  |
| 1 | The article is well written. However, since the authors are working on the factors associated with stroke, a range of the age group taken (Minimum & maximum age in adults) instead of the mean age would be more informative to further identify the incidence of stroke, whether more common in young adults or older adults. | The range of the age group we take is based on previous similar published articles. We searched many articles to get the appropriate age categories finally we believe that this age categories was suitable for our study. In the previous studies age greater than 65 years were high risk for stroke and they used similar categories of age. |
| 2 | The inclusion of some more parameters like Gender, Socioeconomic status, dietary habits (Consumption of more Fatty or non fatty food), profession (Stressful/ non stressful), life style (Sedentary/ Active), & family history will make this study and identification of factors more meaningful. | We assessed sex (Male, Female), dietery habits (Did you eat diet high in fat such as Fatty meal and animal product)(Yes, No), profession(occupation)(Farmer, House wife, Government employee, Non-Government organization employee, Self-employee, Other), physical exercise was assed using two questions (i.e How many days do you do regular physical exercise in a week __and On average how much time do you spend during those exercise in a typical day_) finally it was diactomized in to physically active and phyisically inactive and family history of stroke(Yes, No). The socio economic status of the participants, actualy average montlty income was in our questioner but almost in all the questioner the value was missing it may be due to most patients have no fixed monthly income especially participants from the rular area finally due to high missing value we didn’t consider for analysis. |
|  | **Reviewer#2** |  |
| 1 | Usage of English language is not up to the mark, there are several spelling mistakes, grammatical and typo error in this manuscript. | We accept the comments and we made corrections on the grammatical and punctuation errors throughout the whole document. |
| 2 | There is only one reference of 2018 and no reference of 2019 and the referencing is not as per journal format. | We made uptodated informations in our paper. |
| 3 | Annexure I is only available but other supporting tables are not in readable format. | I accepted your comment. Now tables are within the main manscript and individual tables are removed. We made corrections based on the journal format and in areadable form. |
